# The lungs were on fire: a pilot study of ^18^F-FDG PET/CT in idiopathic-inflammatory-myopathy-related interstitial lung disease

**DOI:** 10.1186/s13075-021-02578-9

**Published:** 2021-07-23

**Authors:** Junyu Liang, Heng Cao, Yinuo Liu, Bingjue Ye, Yiduo Sun, Yini Ke, Ye He, Bei Xu, Jin Lin

**Affiliations:** 1 Department of Rheumatology, The First Affiliated Hospital, Zhejiang University School of Medicine, #79 Qingchun Road, Hangzhou, 310003 Zhejiang Province People’s Republic of China; 2 PET Center, The First Affiliated Hospital, Zhejiang University School of Medicine, #79 Qingchun Road, Hangzhou, 310003 Zhejiang Province People’s Republic of China; 3 Department of Respiratory Diseases, The First Affiliated Hospital, Zhejiang University School of Medicine, #79 Qingchun Road, Hangzhou, 310003 Zhejiang Province People’s Republic of China

**Keywords:** Idiopathic inflammatory myopathy, Interstitial lung disease, PET/CT scan, Dermatomyositis, Polymyositis

## Abstract

**Background:**

Interstitial lung disease (ILD) and its rapid progression (RP) are the main contributors to unfavourable outcomes of patients with idiopathic inflammatory myopathy (IIM). This study aimed to identify the clinical value of PET/CT scans in IIM-ILD patients and to construct a predictive model for RP-ILD.

**Methods:**

Adult IIM-ILD patients who were hospitalized at four divisions of the First Affiliated Hospital, Zhejiang University School of Medicine (FAHZJU), from 1 January 2017 to 31 December 2020 were reviewed. PET/CT scans and other characteristics of patients who met the inclusion and exclusion criteria were collected and analysed.

**Results:**

A total of 61 IIM-ILD patients were enrolled in this study. Twenty-one patients (34.4%) developed RP-ILD, and 24 patients (39.3%) died during follow-up. After false discovery rate (FDR) correction, the percent-predicted diffusing capacity of the lung for carbon monoxide (DLCO%, *P* = 0.014), bilateral lung mean standard uptake value (SUVmean, *P* = 0.014) and abnormal mediastinal lymph node (*P* = 0.045) were significantly different between the RP-ILD and non-RP-ILD groups. The subsequent univariate and multivariate logistic regression analyses verified our findings. A “DLM” model was established by including the above three values to predict RP-ILD with a cut-off value of ≥ 2 and an area under the curve (AUC) of 0.905. Higher bilateral lung SUVmean (*P* = 0.019) and spleen SUVmean (*P* = 0.011) were observed in IIM-ILD patients who died within 3 months, and a moderate correlation was recognized between the two values.

**Conclusions:**

Elevated bilateral lung SUVmean, abnormal mediastinal lymph nodes and decreased DLCO% were significantly associated with RP-ILD in IIM-ILD patients. The “DLM” model was valuable in predicting RP-ILD and requires further validation.

**Supplementary Information:**

The online version contains supplementary material available at 10.1186/s13075-021-02578-9.

## Introduction

Interstitial lung disease (ILD) is a frequent complication in patients with idiopathic inflammatory myopathy, showing a predominantly non-specific interstitial pneumonia (NSIP) pattern in histopathological findings [1–3]. A large proportion of idiopathic-inflammatory-myopathy-related ILD (IIM-ILD) cases are rapidly progressive (RP) and refractory to conventional immunosuppressive therapy, making the clinical management of IIM-ILD challenging [4]. In recent studies, the survival of IIM patients with ILD, RP-ILD in particular, is far from satisfactory. Specifically, the mortality rate of IIM-ILD patients varied from 19.0 to 27.0% during follow-up, and a 3-month mortality rate of over 30.0% was identified for RP-ILD [5–7]. Therefore, it is vital to search for efficient biomarkers or tools for predicting the development and progression of RP-ILD in order to avoid unfavourable outcomes.

Activation and infiltration of immune cells and cytokine release participate in the development of idiopathic pulmonary fibrosis (IPF), connective tissue disease-related ILD (CTD-ILD) and coronavirus disease 2019 (COVID-19) [8–11]. Since elevated ^18^F-fluorodeoxyglucose (FDG) uptake on PET/CT scans can indicate focal immune activation, a series of studies have focused on the clinical value of PET/CT scans in these diseases. In systemic sclerosis-related ILD (SSc-ILD), pulmonary FDG uptake was significantly increased and correlated with the severity of ILD [12, 13]. In addition, elevated pulmonary FDG uptake also predicted the progression of SSc-ILD [13]. Dr Morita reported an IIM-ILD patient who presented with high pulmonary FDG uptake and subsequently died of RP-ILD [14]. Subsequently, two small-sample cohort studies found that FDG uptake (maximum standard uptake value, SUVmax) in the lung was correlated with ILD severity and might predict the occurrence of RP-ILD [15, 16]. However, the sample sizes were too small, and the included organs were only the lungs and muscles. Meanwhile, the correlation between abnormal FDG uptake and the survival of IIM-ILD patients remains unclear. A systemic evaluation of FDG uptake in IIM-ILD patients is necessary to acquire a broader view of the clinical value of PET/CT scans in IIM-ILD.

A systemic evaluation of FDG uptake in a larger cohort of IIM-ILD patients was thus implemented to clarify the predictive value of FDG uptake in multiple organs for RP-ILD as well as its ability to predict unfavourable outcomes. The mean value of the region of interest (ROI) instead of the maximum value at a single pixel was calculated to avoid interference and to acquire a more representative FDG uptake of the targeted organ. In addition, we constructed a model to predict RP-ILD and unfavourable outcomes in IIM-ILD patients by incorporating statistically significant values on PET/CT scans, lung function testing, and other variables.

## Patients and methods

### Patients

A retrospective cohort study was conducted at the inpatient department of Qingchun, Chengzhan, Zhijiang and Yuhang divisions of the First Affiliated Hospital, Zhejiang University School of Medicine (FAHZJU). After acquiring approval from the Institutional Review Board (IRB) of FAHZJU (Reference Number: 2021-194) and written informed consent from all of the patients involved, in accordance with the Declaration of Helsinki, a case search was retrospectively conducted of the inpatient electronic medical record (EMR) system for patients with discharge diagnoses of dermatomyositis (DM), polymyositis (PM) or amyopathic dermatomyositis (ADM), from 1 January 2017 to 31 December 2020. The inclusion criteria for this study were (1) age over 18 years old; (2) a definite/probable diagnosis of DM, PM or ADM applying the 2017 ACR/EULAR classification criteria, as confirmed by two experienced rheumatologists (Heng Cao and Bei Xu) [17]; (3) ILD on high-resolution computed tomography (HRCT) within the first week of admission, including the usual interstitial pneumonia (UIP) pattern and non-UIP patterns (NSIP, cryptogenic organizing pneumonia, the coexistence of more than one CT pattern), as confirmed by an experienced radiologist and a respiratory specialist (Yinuo Liu and Bingjue Ye); and (4) a PET/CT scan performed during hospitalization. The exclusion criteria were (1) clarified overlap syndromes with other connective tissue diseases (CTDs); (2) myopathy related to thyroid dysfunction, strenuous exercise, inherited metabolic disorders, drug-induced myositis (lamivudine, statins, Chinese herbal medicine, etc.; (3) hospitalization for reasons unrelated to myositis and its complications, such as fracture, pregnancy, acquired immunodeficiency syndrome, cataract, etc.; (4) newly identified or unresolved malignancies; and (5) loss to follow-up without death from any cause within 3 months after hospitalization.

### Data collection

Data from all of the enrolled patients were retrospectively collected by referring to the EMR system of FAHZJU. Clinical records, including demographic information, the course of the disease, the duration of diagnostic delay, disease activity assessment, complications, radiological/laboratory results, lung function testing, and immunosuppressive medications, were acquired and analysed. Survival data were acquired from the follow-up data. Specifically, IIM patients were followed from hospitalization until the end of the follow-up period or death, whichever happened first. For patients who died during hospitalization, their dates of death were clearly documented in the EMR system. For patients who were discharged, a routine return visit was arranged 2 weeks after discharge. In addition to the regular inpatient or outpatient visits, a brief telephone interview was performed 3 months after discharge and then every year. The end of follow-up could be due to death from any cause, loss to follow-up or closure of follow-up for the purpose of this study (31 March 2021).

Baseline disease activity assessment, laboratory tests, lung HRCT scans and lung function testing were carried out within the first week of hospitalization. On-admission IIM disease activity was routinely assessed using the Myositis Disease Activity Assessment Visual Analogue Scale (MYOACT) [18]. ILD and its rapid progression were confirmed by experienced respiratory specialist and radiologist (Bingjue Ye and Yinuo Liu) using lung HRCTs. Cases with definite or probable UIP patterns were identified based on their classic HRCT manifestation: the presence of basal-dominant reticular opacities and a predominantly basal and subpleural distribution of honeycomb lesions, with multiple equal-sized cystic lesions of two to ten mm diameter with a thick wall [19].

A subgroup of RP-ILD patients was considered who presented with progressive dyspnoea and progressive hypoxemia, with acute worsening of interstitial changes on chest radiographs within 1 month after hospitalization or the onset of respiratory symptoms [20–22]. The included IIM-ILD patients were divided into an RP-ILD group and a non-RP-ILD group (control group). Since pulmonary infection is easily confused with interstitial lung disease, the identification of bacterial, fungal, or tuberculosis infection requires a careful and comprehensive investigation based on the essential microbiological findings in sputum or blood, the clinical manifestations, and the radiographic and laboratory abnormalities. In addition, the diagnosis of Epstein-Barr virus (EBV) and cytomegalovirus (CMV) infection relied on the detection of serum antibodies and DNA.

All of the included patients received potent immunosuppressive medications: (1) systemic prednisolone (PSL) or methylprednisolone (mPSL) with a maximum dosage ≥ 1 mg/kg/day (calculated for prednisolone) and (2) combined therapy of PSL/mPSL, disease-modifying anti-rheumatic drugs (DMARDs) or Janus kinase (JAK) inhibitors, with or without intravenous immunoglobulin (IVIG). The DMARDs used for these patients included mycophenolate, tacrolimus, cyclosporine, methotrexate, thalidomide, hydroxychloroquine and cyclophosphamide. JAK inhibitors mainly referred to tofacitinib and baricitinib.

The profiles of 12 myositis-specific antibodies (MSAs, anti-MDA5, anti-TIF1γ, anti-Jo-1, anti-OJ, anti-EJ, anti-PL-12, anti-PL-7, anti-Mi-2α, anti-Mi-2β, anti-NXP2, anti-SRP and anti-SAE1) and four myositis-associated antibodies (MAAs, anti-Ku, anti-PM-Scl75, anti-PM-Scl100 and anti-Ro-52) were assessed by an immunoblotting assay utilizing the EUROLINE Autoimmune Inflammatory Myopathies 16 Ag (IgG) commercial line blot assay (Euroimmun, Lübeck, Germany) encompassing a membrane strip with the 16 autoantigens following the manufacturer’s instructions. Serum samples were routinely acquired from suspected IIM patients within the first week of hospitalization and subjected to testing.

Whole-body CT and PET, which were performed with a combined PET/CT scanner (Biograph, Sensation 16, Siemens systems), covered a region ranging from the meatus of the ear to the mid-thigh. Patients fasted overnight or for at least 6 h prior to PET/CT detection. Blood glucose levels were confirmed to be within normal limits before the injection of 4.0 MBq/kg [18F]FDG. Patients rested for 30 min to minimize non-specific FDG uptake by muscles. Imaging acquisition was systematically implemented at 60 min post-injection. SUV (standard uptake value) was calculated by the following formula: SUV (g/ml) = regional radioactivity concentration (Bq/ml)/[injected dose (Bq)/body weight (g)]. The ROI (20 mm diameter) was manually placed by a single trained radiologist (Yinuo, Liu) in the region with the highest FDG uptake in the liver, spleen, and bone marrow (thoracic, T10-12, lumbar, L2-L4) [23], oesophagus, stomach, small intestine, colon/rectum, bilateral lung, bilateral cerebellum, bilateral proximal muscles (namely, trapezius, deltoid, biceps, iliopsoas, gluteus medius, gluteus maximus and quadriceps) [24], excluding the regions prominently influenced by FDG uptake in other anatomical structures. To avoid noise and acquire the value representing a certain volume of the targeted organs, SUV was calculated as the mean value of ROI (SUVmean) instead of the maximum value at a single pixel [25]. For bilaterally distributed organs, the SUVmean was documented as the maximum SUVmean value of the symmetrical sides. Abnormal hilar or mediastinal lymph nodes were defined as those with swelling and elevated FDG uptake. The radiologist was blinded to the complications and outcome of the included patients when evaluating the SUVmean value of each and every targeted organ.

### Statistical analysis

Statistical analysis was performed using SPSS 22.0 (Chicago*,* IL*,* USA), GraphPad Prism 8.0 and R 3.6.1. An independent sample *t* test was utilized to compare normally distributed continuous variables between the RP-ILD and control groups. The Mann-Whitney *U* test was used to compare skewed continuous variables or ordinal categorical variables. The chi-square test and Fisher’s exact test were applied to compare unordered categorical variables. Logistic regression analyses were used to further verify clinical factors significantly associated with RP-LD. Survival in different groups was assessed by the Kaplan-Meier method with the log-rank test. Cox proportional hazards regression analyses were subsequently adopted to identify the effect of clinical factors on the time to death from any cause. *P* values of the comparisons and univariate analyses were adjusted by false discovery rate (FDR) correction, utilizing the p.adjust function in R.3.6.1, to acquire adjusted *p* values and to minimize type I error. Explanatory factors with *P* < 0 .05 in the univariate analyses were entered into the subsequent multivariate analyses. The correlation between two continuous variables was quantified with Pearson linear analysis. Receiver operating characteristic (ROC) curve analysis was performed to evaluate the predictive value of the continuous variables. All tests were two-sided, and *P* <  0.05 was deemed statistically significant.

## Results

**Fig. 1:**
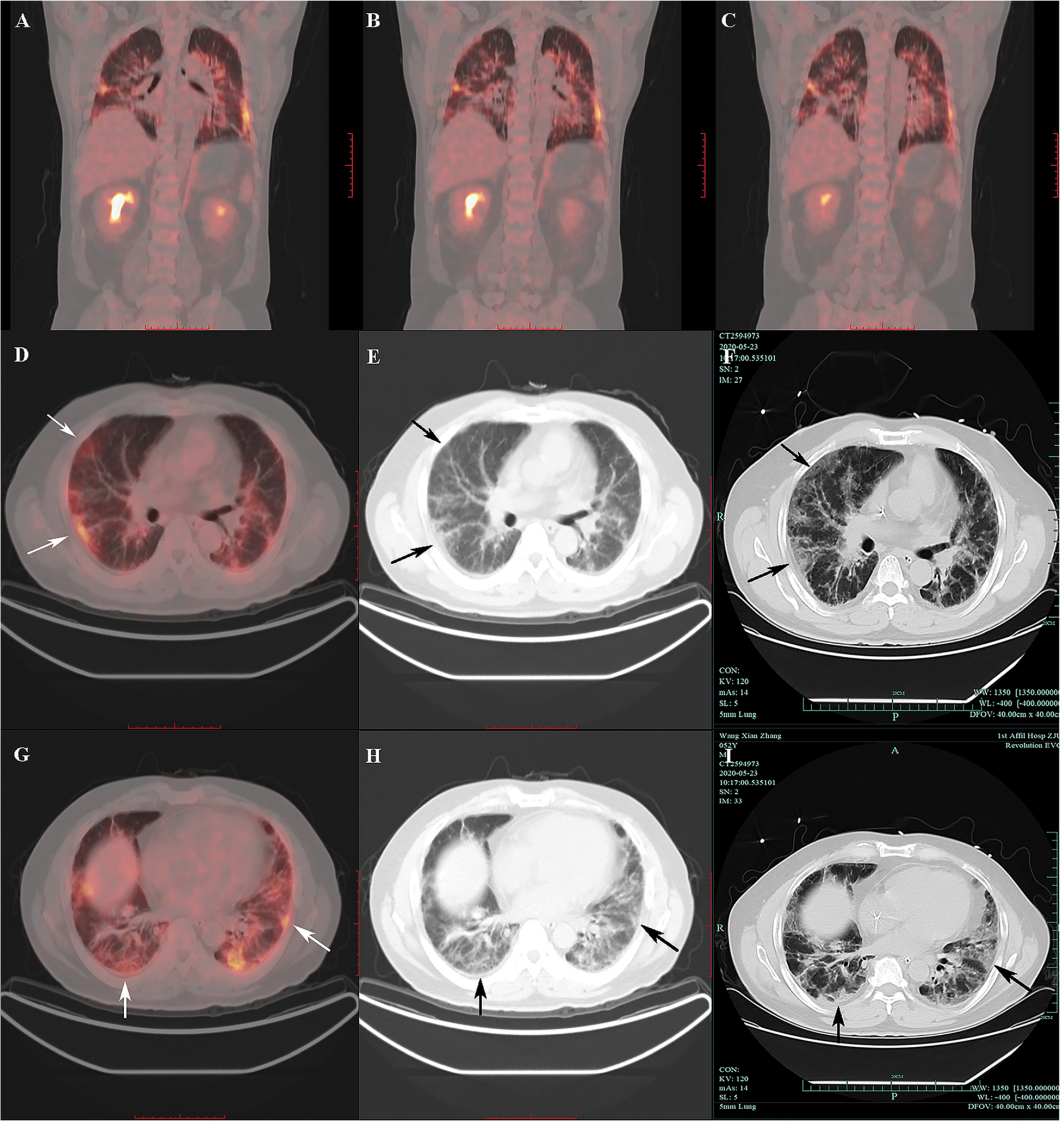
Visual examination of ^18^F-FDG-PET/CT scan and HRCT of one IIM-ILD patients who developed RP-ILD. **A** to **C**. Prominently elevated FDG uptake in bilateral lungs of one IIM-ILD patient who later developed RP-ILD. **D** to **F** and **G** to **I**. Prominently elevated FDG uptake in bilateral lungs of one IIM-ILD patient indicated future RP-ILD in one week (where the arrow pointed). FDG: Fluorodeoxyglucose; HRCT: High resolution CT; IIM-ILD: Idiopathic-inflammatory-myopathy-related interstitial lung disease; RP-ILD: Rapidly progressive interstitial lung disease

**Fig. 2:**
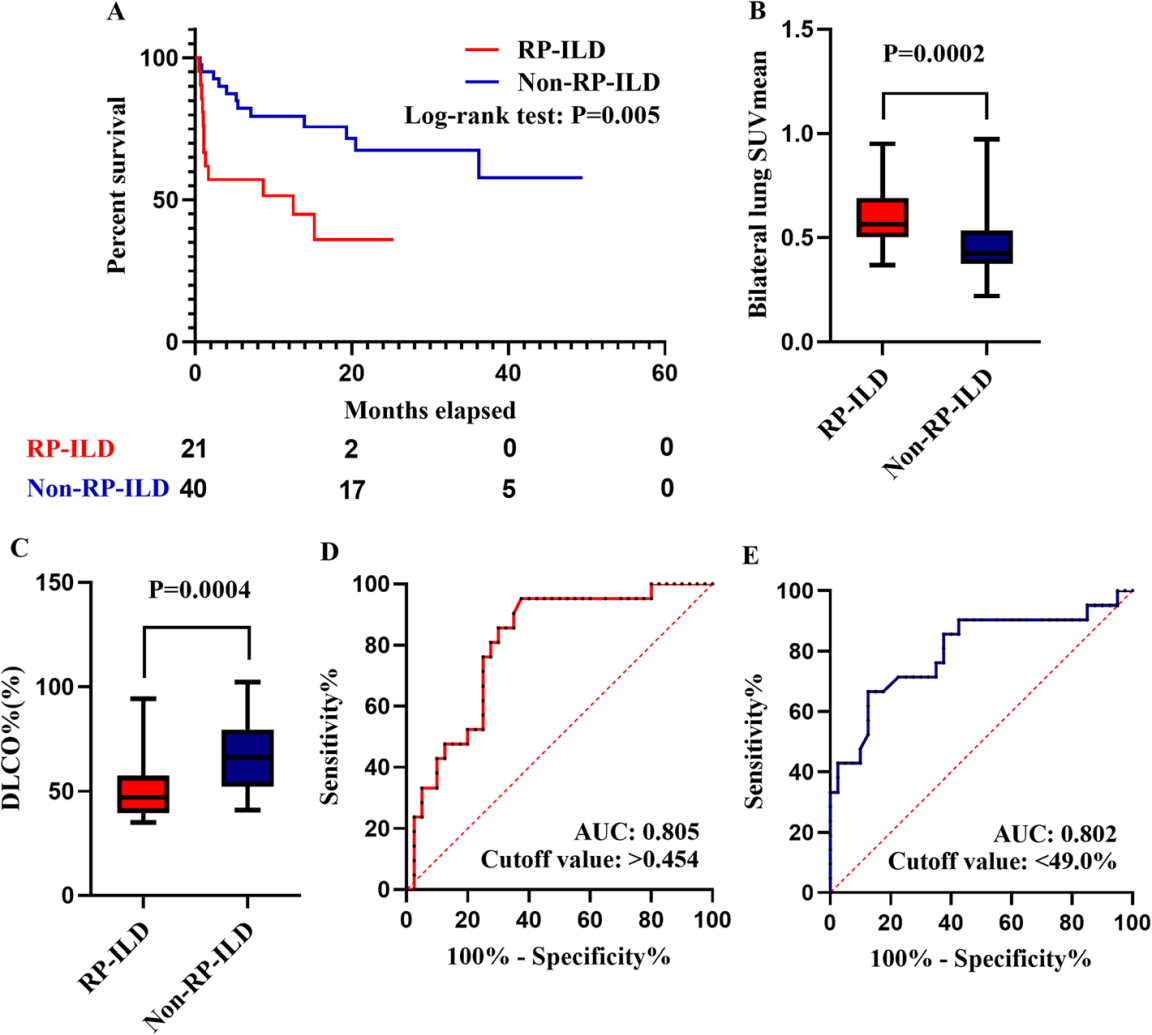
Evaluation of IIM-ILD patients with or without RP-ILD. **A**. Survival of IIM-ILD patients with or without RP-ILD. **B**. Comparison of bilateral lung SUVmean in RP-ILD and non-RP-ILD groups. **C**. Comparison of DLCO% in RP-ILD and non-RP-ILD groups. **D**. ROC curve of bilateral lung SUVmean predicting RP-ILD. **E**. ROC curve of DLCO% predicting RP-ILD. IIM-ILD: Idiopathic-inflammatory-myopathy-related interstitial lung disease; RP-ILD: Rapidly progressive interstitial lung disease; SUVmean: mean standard uptake value; DLCO%: Percent-predicted diffusing capacity of the lung for carbon monoxide; ROC: Receiver operating characteristic; AUC: Area under the curve

From 1 January 2017 to 31 December 2020, 274 adult IIM-ILD patients were admitted to the Qingchun, Chengzhan, Zhijiang and Yuhang divisions of FAHZJU. Among them, 61 patients who satisfied the inclusion/exclusion criteria were included in the study (Additional file 1), encompassing 40 with DM, nine with PM and 12 with ADM. Their mean age was 56.72 ± 11.28 years old and 25 were men (41.0%). Twenty-four patients (39.3%) died during follow-up, and the median follow-up time was 11.90 (4.00, 23.80) months. Among the included patients, 21 patients (34.4%) developed RP-ILD (Fig. 1). The other 40 patients without RP-ILD constituted the control group. All of the RP-ILD events were identified after taking PET/CT scans. IIM patients with RP-ILD were found to suffer from shorter survival (*P* = 0.005, Fig. 2A), with 9 RP-ILD patients (42.9%) dying within 3 months after hospitalization.

An unadjusted comparison between IIM-ILD patients with or without RP-ILD showed that patients who later developed RP-ILD had more complications of pulmonary bacterial infection (*P* = 0.036) and gastrointestinal haemorrhage (*P* = 0.044), higher MYOACT score (*P* = 0.006), a higher bilateral lung SUVmean (*P* < 0.001, Fig. 2B), more abnormal mediastinal (*P* = 0.002) and hilar (*P* = 0.018) lymph nodes, lower total lung capacity (TLC, *P* = 0.049), lower percent-predicted diffusing capacity of the lung for carbon monoxide (DLCO%, *P* < 0.001, Fig. 2C) and positivity of anti-MDA5 antibody (*P* = 0.016). However, after FDR correction, only DLCO% (*P* = 0.014), bilateral lung SUVmean (*P* = 0.014) and abnormal mediastinal lymph nodes (*P* = 0.045) remained significant (Table 1). The subsequent univariate and multivariate logistic regression analyses also revealed the statistical significance of DLCO% (*P* = 0.003), bilateral lung SUVmean (*P* = 0.001) and abnormal mediastinal lymph nodes (*P* = 0.013) in association with RP-ILD (Additional files 2 and 3). No significant correlation was identified among the three clinical factors (*P* = 0.491 and *r* = − 0.090 for bilateral lung SUVmean and DLCO%, *P* = 0.243 for bilateral lung SUVmean and abnormal mediastinal lymph node, *P* = 0.077 for DLCO% and abnormal mediastinal lymph node, Additional file 4).

**Table 1:** Comparisons of multiple factors between RP-ILD and non-RP-ILD groups

Factors	RP-ILD (21)	Non-RP-ILD (40)	***P*** value	***P***-adjusted
**Age(y)**	**54.86 ± 11.72**	**57.70 ± 11.06**	**0.354**	**0.824**
**Sex (male/female)**	**8/13**	**17/23**	**0.740**	**1.000**
**Course of disease(m)**	**2.00 (1.00, 2.75)**	**2.25 (1.00, 5.00)**	**0.253**	**0.748**
**Duration of diagnosis delay(m)**	**1.50 (0.50, 2.00)**	**1.00 (1.00, 3.00)**	**0.274**	**0.756**
**Clinical manifestations or complications**				
**Pulmonary bacterial infection**	**7 (33.3%)**	**4 (10.0%)**	**0.036**	**0.347**
**Pulmonary fungal infection**	**5 (23.8%)**	**4 (10.0%)**	**0.253**	**0.748**
**Tuberculosis infection**	**0 (0.0%)**	**0 (0.0%)**	**NA**	**NA**
**EBV infection**	**6 (28.6%)**	**5 (12.5%)**	**0.164**	**0.605**
**CMV infection**	**2 (9.5%)**	**0 (0.0%)**	**0.115**	**0.559**
**Carcinoma**	**1 (4.8%)**	**6 (15.0%)**	**0.405**	**0.824**
**Gastrointestinal haemorrhage**	**4 (19.0%)**	**1 (2.5%)**	**0.044**	**0.347**
**UIP pattern**	**3 (14.3%)**	**4 (10.0%)**	**0.683**	**0.962**
**Pneumomediastinum**	**1 (4.8%)**	**1 (2.5%)**	**1.000**	**1.000**
**Laboratory finding**				
**Ferritin (ng/ml)**	**612.50 (302.05, 2110.25)**	**664.05 (314.40, 2061.23)**	**0.495**	**0.863**
**ESR (mm/h)**	**16.00 (6.50, 32.00)**	**17.50 (9.50, 42.00)**	**0.466**	**0.857**
**CRP (mg/L)**	**5.40 (3.10, 19.80)**	**6.35 (3.00, 36.73)**	**0.660**	**0.962**
**ALT(U/L)**	**37.00 (22.50, 99.50)**	**91.50 (33.75, 212.50)**	**0.051**	**0.347**
**AST(U/L)**	**45.00 (33.00, 97.00)**	**54.50 (34.00, 235.50)**	**0.224**	**0.725**
**LDH(U/L)**	**329.00 (275.50, 478.50)**	**323.00 (247.75, 461.50)**	**0.897**	**1.000**
**CK(U/L)**	**84.00 (50.50, 255.00)**	**86.00 (44.25, 415.25)**	**0.838**	**1.000**
**Disease activity**				
**MYOACT score**	**13.00 (9.00, 15.50)**	**9.00 (7.00, 10.75)**	**0.006**	**0.102**
**Lung function testing**				
**FVC% (%)**	**65.33 ± 20.08**	**71.86 ± 15.61**	**0.165**	**0.605**
**FEV1% (%)**	**64.85 ± 19.40**	**74.42 ± 18.25**	**0.062**	**0.383**
**FEV1/FVC**	**0.81 (0.75, 0.85)**	**0.81 (0.77,0.85)**	**0.849**	**1.000**
**TLC(L)**	**3.43 ± 1.01**	**3.98 ± 1.02**	**0.049**	**0.347**
**DLCO% (%)**	**50.29 ± 15.58**	**66.92 ± 16.67**	**< 0.001**	**0.014**
^**18**^**F-FDG PET/CT scan findings**				
Time gap after RS onset*(d)	**20.00 (12.50, 25.00)**	**16.50 (13.00, 24.75)**	**0.767**	**1.000**
**Bilateral lung SUVmean**	**0.60 ± 0.15**	**0.45 ± 0.14**	**< 0.001**	**0.014**
**Abnormal mediastinal lymph node**	**14 (66.7%)**	**10 (25.0%)**	**0.002**	**0.045**
**Abnormal hilar lymph node**	**11 (52.4%)**	**9 (22.5%)**	**0.018**	**0.204**
**Liver SUVmean**	**1.83 ± 0.34**	**1.73 ± 0.47**	**0.412**	**0.824**
**Spleen SUVmean**	**2.02 ± 0.50**	**1.82 ± 0.46**	**0.126**	**0.571**
**Bone marrow SUVmean**	**1.72 ± 0.50**	**1.62 ± 0.44**	**0.402**	**0.824**
**Cardiac SUVmean**	**1.38 (1.00, 2.03)**	**1.60 (1.12, 2.39)**	**0.379**	**0.824**
**Oesophagus SUVmean**	**1.51 (1.19, 1.83)**	**1.34 (1.07, 1.74)**	**0.213**	**0.724**
**Stomach SUVmean**	**0.69 (0.49, 1.00)**	**0.71 (0.57, 0.83)**	**0.927**	**1.000**
**Small intestine SUVmean**	**1.05 ± 0.33**	**1.03 ± 0.32**	**0.878**	**1.000**
**Colon and rectum SUVmean**	**1.07 (0.80, 1.32)**	**1.02 (0.83, 1.20)**	**0.693**	**0.962**
**Bilateral cerebellum SUVmean**	**5.17 ± 1.26**	**5.24 ± 1.61**	**0.863**	**1.000**
**Bilateral trapezius SUVmean**	**0.82 (0.70, 0.96)**	**0.79 (0.68, 1.00)**	**0.585**	**0.944**
**Bilateral deltoid SUVmean**	**0.71 (0.58, 1.27)**	**0.78 (0.61, 1.09)**	**0.590**	**0.944**
**Bilateral biceps SUVmean**	**0.75 (0.67, 1.01)**	**0.77 (0.60, 1.10)**	**0.879**	**1.000**
**Bilateral iliopsoas SUVmean**	**1.10 ± 0.36**	**1.12 ± 0.36**	**0.850**	**1.000**
**Bilateral gluteus maximus SUVmean**	**0.85 ± 0.27**	**0.79 ± 0.32**	**0.462**	**0.857**
**Bilateral gluteus medius SUVmean**	**0.92 ± 0.29**	**0.98 ± 0.29**	**0.473**	**0.857**
**Bilateral quadriceps SUVmean**	**0.78 (0.62, 0.96)**	**0.82 (0.69, 1.05)**	**0.370**	**0.824**
**Myositis-specific antibodies and myositis-associated antibodies**				
**Anti-MDA5**	**13 (61.9%)**	**12 (30.0%)**	**0.016**	**0.204**
**Anti-PL-7**	**4 (19.0%)**	**2 (5.0%)**	**0.169**	**0.605**
**Anti-PL-12**	**1 (4.8%)**	**1 (2.5%)**	**1.000**	**1.000**
**Anti-EJ**	**0 (0.0%)**	**1 (2.5%)**	**1.000**	**1.000**
**Anti-OJ**	**0 (0.0%)**	**1 (2.5%)**	**1.000**	**1.000**
**Anti-Jo-1**	**2 (9.5%)**	**3 (7.5%)**	**1.000**	**1.000**
**Anti-TIF1γ**	**1 (4.8%)**	**3 (7.5%)**	**1.000**	**1.000**
**Anti-Mi-2α**	**0 (0.0%)**	**2 (5.0%)**	**0.541**	**0.920**
**Anti-Mi-2β**	**0 (0.0%)**	**4 (10.0%)**	**0.289**	**0.756**
**Anti-SAE1**	**0 (0.0%)**	**5 (12.5%)**	**0.154**	**0.605**
**Anti-NXP2**	**1 (4.8%)**	**6 (15.0%)**	**0.405**	**0.824**
**Anti-SRP**	**1 (4.8%)**	**2 (5.0%)**	**1.000**	**1.000**
**Anti-Ku**	**1 (4.8%)**	**1 (2.5%)**	**1.000**	**1.000**
**Anti-PM-Scl75**	**1 (4.8%)**	**2 (5.0%)**	**1.000**	**1.000**
**Anti-PM-Scl100**	**0 (0.0%)**	**0 (0.0%)**	**NA**	**NA**
**Anti-Ro-52**	**14 (66.7%)**	**17 (42.5%)**	**0.073**	**0.414**
**Therapies**				
**Steroid monotherapy**	**6 (28.6%)**	**14 (35.0%)**	**0.611**	**0.944**
**Steroid + DMARDs**	**7 (33.3%)**	**19 (47.5%)**	**0.288**	**0.756**
**Steroid+IVIG**	**2 (9.5%)**	**2 (5.0%)**	**0.602**	**0.944**
**Steroid+DMARDs+IVIG**	**3 (14.3%)**	**4 (10.0%)**	**0.683**	**0.962**
**Steroid + JAK inhibitor**	**3 (14.3%)**	**1 (2.5%)**	**0.113**	**0.559**
**IIM subtypes**				
**DM**	**13 (61.9%)**	**27 (67.5%)**	**0.662**	**0.962**
**PM**	**2 (9.5%)**	**7 (17.5%)**	**0.479**	**0.857**
**ADM**	**6 (28.6%)**	**6 (15.0%)**	**0.309**	**0.778**

RP-ILD, rapidly progressive interstitial lung disease; *P*-adjusted, adjusted *P* value after false discovery rate correction; y, years; m, months; NA, not available; EBV, Epstein-Barr virus; CMV, cytomegalovirus; UIP pattern, usual interstitial pneumonia pattern; ESR, erythrocyte sedimentation rate; CRP, C-reactive protein; ALT, alanine transaminase; AST, aspartate transaminase; LDH, lactate dehydrogenase; CK, creatine kinase; MYOACT, Myositis Disease Activity Assessment Visual Analogue Scales; FVC%, percent-predicted forced vital capacity; FEV1%, percent-predicted forced expiratory volume in 1 s; FEV1/FVC, ratio of FEV1 over FVC; TLC, total lung capacity; DLCO%, percent-predicted diffusing capacity of the lung for carbon monoxide; RS, respiratory symptoms; d, days; FDG, fluorodeoxyglucose; SUVmean, mean standard uptake value; DMARDs, disease-modifying anti-rheumatic drugs; IVIG, intravenous immunoglobulin; JAK, Janus kinase; IIM, idiopathic inflammatory myopathy; DM, dermatomyositis; PM, polymyositis; ADM, amyopathic dermatomyositis

*Time gap after RS onset referred to the time gap between onset of respiratory symptoms (evident feelings of chest distress and shortness of breath) and PET/CT scan

Utilizing ROC curve analysis, the optimal cut-off value of the bilateral lung SUVmean for RP-ILD was > 0.454, with a sensitivity of 95.2% and a specificity of 62.5%. The area under the curve (AUC) was 0.805 (Fig. 2D). Meanwhile, the optimal cut-off value of DLCO% for RP-ILD was < 49.0%, with a sensitivity of 87.5%, a specificity of 66.7% and an AUC of 0.802 (Fig. 2E). Moreover, pulmonary bacterial infection (*P* = 0.007) and pulmonary fungal infection (*P* = 0.034) were associated with elevated bilateral lung SUVmean. A moderate correlation was also recognized between the MYOACT score (*P* = 0.004, *r* = 0.360) and the bilateral lung SUVmean. Nevertheless, no correlation was identified between the bilateral lung SUVmean and the time gap after the onset of respiratory symptoms (*P* = 0.395, *r* = -0.111) (Additional file 5).

**Fig. 3:**
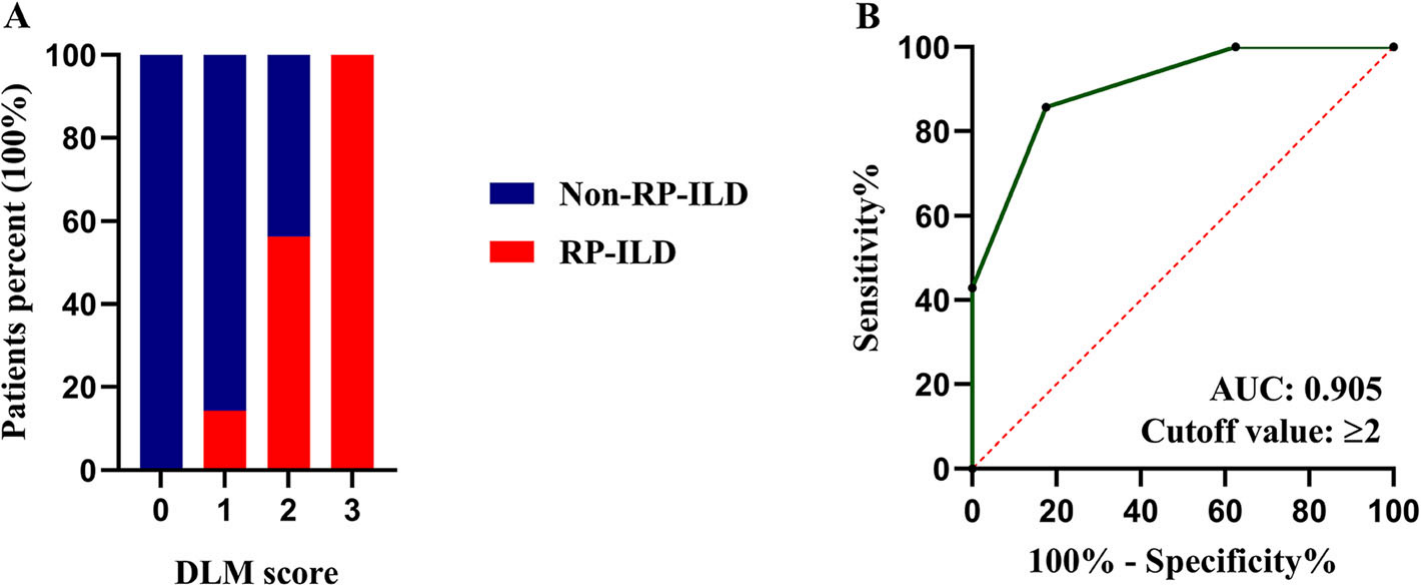
Evaluation of DLM model in predicting RP-ILD in IIM-ILD patients. **A**. Distribution of RP-ILD and non-RP-ILD patients in each DLM score group. **B**. ROC curve of DLM model predicting RP-ILD. RP-ILD: Rapidly progressive interstitial lung disease; IIM-ILD: Idiopathic-inflammatory-myopathy-related interstitial lung disease; ROC: Receiver operating characteristic; AUC: Area under the curve

To develop a scoring system for the prediction of RP-ILD, we rounded up the cut-off values of bilateral SUVmean and DLCO% to > 0.450 and < 50.0%, respectively. Each was allotted one point. The identification of abnormal mediastinal lymph nodes was also allotted one point. Each included patient was then assigned a cumulative score (maximum score of three, minimum score of zero). The scoring system was named “DLM” using the initials of DLCO%, lung and mediastinum. Then, the predictive value of the DLM model was evaluated in 61 patients. None of the 15 patients with the minimum possible score of zero developed RP-ILD. In contrast, all nine patients with a maximum possible score of three developed RP-ILD (Table 2, Fig. 3A). In the ROC curve analysis (Fig. 3B), we found a cut-off value of ≥ 2, AUC of 0.905, sensitivity of 85.7%, specificity of 82.5%, positive predictive value (PPV) of 72.0%, negative predictive value (NPV) of 91.7% and accuracy of 83.6%. Furthermore, the predictive value of the DLM model was verified in univariate and multivariate logistic regression analyses for RP-ILD in IIM-ILD patients. After incorporating the DLM score into the statistical model, the DLM score was significantly correlated with RP-ILD with *P* < 0.001, and all of the other factors were excluded from the model (Additional file 6).

**Table 2:** Distribution of RP-ILD and non-RP-ILD in IIM-ILD patients with different DLM score

DLM score	Total patients (N)	RP-ILD % (N)	Non-RP-ILD % (N)
**0**	**15**	**0.0% (0)**	**100.0% (15)**
**1**	**21**	**14.3% (3)**	**85.7% (18)**
**2**	**16**	**56.3% (9)**	**43.7% (7)**
**3**	**9**	**100.0% (9)**	**0.0% (0)**

RP-ILD, rapidly progressive interstitial lung disease; IIM-ILD, idiopathic-inflammatory-myopathy-related interstitial lung disease; N, number

**Fig. 4:**
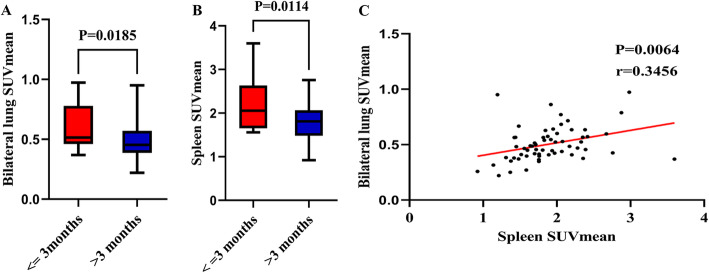
Evaluation of abnormal FDG uptake in IIM-ILD patients with different survival. **A**. Comparison of bilateral lung SUVmean in IIM-ILD patients who died within three months or survived beyond this threshold. **B**. Comparison of spleen SUVmean in IIM-ILD patients who died within three months or survived beyond three months. **C**. Correlation between bilateral lung SUVmean and spleen SUVmean in IIM-ILD patients. FDG: Fluorodeoxyglucose; IIM-ILD: Idiopathic-inflammatory-myopathy-related interstitial lung disease; SUVmean: mean standard uptake value

To explore the predictive value of PET/CT scans for survival, a univariate Cox proportional hazards regression analysis identified pulmonary bacterial infection (*P* < 0.001), RP-ILD (*P* = 0.008), MYOACT score (*P* < 0.001), DLCO% (*P* = 0.016), bilateral lung SUVmean (*P* = 0.007), spleen SUVmean (*P* = 0.008) and the use of steroid+IVIG (*P* = 0.015) as significantly correlated with survival during follow-up (Additional file 7). The subsequent multivariate Cox proportional hazards regression analysis identified pulmonary bacterial infection (*P* = 0.013) and MYOACT score (*P* < 0.001) as factors significantly related to the survival of IIM-ILD patients (Additional file 8). Furthermore, IIM-ILD patients who died within 3 months were found to have a higher bilateral lung SUVmean (*P* = 0.019, Fig. 4A) and a higher spleen SUVmean (*P* = 0.011, Fig. 4B). In addition, a moderate correlation was recognized between spleen SUVmean and bilateral lung SUVmean (*P* = 0.006, *r* = 0.346, Fig. 4C). Nevertheless, in the RP-ILD subgroups, bilateral lung SUVmean (*P* = 0.598, Additional file 9) and spleen SUVmean (*P* = 0.161, Additional file 9) were not recognized to be significantly different between patients who died within 3 months or survived beyond this threshold.

## Discussion

Apart from its uses in oncologic imaging, ^18^F-FDG PET/CT has also been found to reflect immune cell activation in the spleen, malignant tissues and lungs [25, 26]. In a bleomycin-induced pulmonary fibrosis mouse model, the early stage of FDG uptake increase was probably related to the early recruitment and activation of leukocytes, while the later and persistent elevation of FDG uptake might be associated with aerobic glycolysis of myofibroblasts [12, 27, 28]. Furthermore, elevated pulmonary FDG uptake was also found to be associated with macrophage and neutrophil activation in acute or chronic lung diseases [29, 30]. Elevated ^18^F-FDG uptake could therefore serve as a marker for focal and global inflammatory activation and elevated metabolism in bilateral lungs. In addition, two antifibrotic drugs, namely, nintedanib and pirfenidone, were found to significantly decrease pulmonary FDG uptake in bleomycin-treated mice, indicating the value of PET/CT scans in evaluating the response to antifibrotic medications [31, 32].

However, the role of PET/CT scans requires further validation in IPF and CTD-ILD patients. To the best of our knowledge, this is the largest cohort to investigate the clinical value of pulmonary FDG uptake in IIM-ILD patients and the first study to systemically explore FDG uptake in extra-pulmonary organs (liver, spleen, digestive tract, etc.) in IIM-ILD patients.

After initially confirming the role of FDG uptake in reflecting focal immune and metabolic activation, we used a retrospective cohort to identify the clinical value of PET/CT scans for RP-ILD in IIM-ILD patients. Both increased pulmonary FDG uptake and abnormal mediastinal lymph nodes were significantly correlated with RP-ILD in IIM-ILD patients. Compared with conventional HRCT, PET/CT scans seemed to be more intuitive for predicting RP-ILD. In IPF patients, PET/CT scans have been widely studied and found to be valuable for the evaluation of disease activity and therapy efficacy as well as the prediction of disease progression and survival [31, 33, 34]. In patients with lung cancer, elevated pulmonary FDG uptake is associated with acute exacerbation of ILD after chemotherapy or surgery [35–37]. In Ssc-ILD patients, pulmonary FDG uptake is correlated with ILD severity [13]. Together with abnormal mediastinal/hilar lymph nodes, it is associated with the progression of ILD within 2 years [13]. The predictive value of pulmonary FDG uptake for ILD progression crossed the boundary of IPF, tumour-associated ILD and CTD-ILD and needs to be verified with a broader ILD spectra and larger cohorts. In addition, in our study, elevated pulmonary FDG uptake was associated with pulmonary bacterial/fungal infection and elevated IIM disease activity. These findings reflect the focal immune abnormality of both IIM and an infectious background, consistent with previous reports that CTD-related immune alterations and infectious triggers played important roles in the progressive phenotype of CTD-ILD [38, 39].

Before FDR correction, pulmonary and splenic FDG uptake was found to be correlated with the survival of IIM-ILD patients. The outcome-predicting value of pulmonary FDG uptake has been previously proposed for IPF [40]. Fraioli and his colleagues proposed that including pulmonary FDG uptake in the conventional GAP (gender, age and physiology) model would significantly increase the model’s accuracy for outcome prediction [41]. Through Pearson linear analysis, we also found a moderate correlation between the spleen SUVmean and bilateral lung SUVmean. As the largest lymphoid organ containing specific subsets of myeloid cells and lymphocytes, the spleen is involved in CTD development [42]. We hypothesized that there might be a link between the spleen and lung during the development of CTD-ILD. In traditional Chinese medicine, the spleen is the organ that produces and replenishes Qi, which tonifies and nourishes the lung [43]. It will be interesting and meaningful to identify cross-talk between the spleen and lung from the perspective of modern medicine. However, only higher disease activity and complications of pulmonary infection remained statistically significant after FDR correction and multivariate analysis, which was consistent with the findings of previous studies [44, 45].

RP-ILD is a severe and fatal complication of IIM patients, and it is thus necessary to search for early prediction tools to enable the application of timely and potent therapeutic regimens for patients at high risk of RP-ILD. Previous studies indicated that anti-MDA5 antibody, anti-Ro52 antibody, DLCO%, serum ferritin, etc., were correlated with the development of RP-ILD [46, 47]. After FDR correction and multivariate logistic regression analysis, bilateral lung SUVmean, abnormal mediastinal lymph node and DLCO% were significantly different between patients with RP-ILD and the control group. The three clinical factors were tested independently and were then incorporated to form the DLM model. The DLM model had satisfying AUC, sensitivity, specificity, PPV, NPV and accuracy. However, due to the scarcity of IIM-ILD patients receiving PET/CT scans, we could not construct another cohort of IIM-ILD patients to validate the DLM score.

There were multiple limitations of this study. In this small-sample study, selection bias occurred since patients with fever, signs of cytopenia, higher disease activity and dyspnoea tended to receive PET/CT scans to exclude malignancy or a relapse of malignancy. The proportion of anti-MDA5 antibody-positive patients was thus higher than that reported previously, which might impede the identification of the role of anti-MDA5 antibody in RP-ILD and survival. Due to its retrospective nature, the small sample size and the high heterogeneity of the disease profile, we could not study additional clinical factors, such as Kreb Von Den Lungen-6 and the HRCT score. The lack of timely follow-up lung function testing in multiple patients made it impossible to incorporate deterioration of lung function into the criteria of RP-ILD. The scarcity of IIM patients receiving PET/CT scans made it impossible to verify the predictive value of the DLM model in a validation cohort. Healthy people or IIM patients without ILD seldom receive PET/CT scans, making it impossible to construct a healthy control group or select IIM patients without ILD as controls. To make the prediction model easier to construct, the bilateral lung SUVmean was calculated at one site with the highest FDG uptake instead of multiple sites. Despite all of these limitations, we were able to identify the clinical value of PET/CT scans in IIM-ILD and constructed a scoring system for the prediction of RP-ILD, and our findings suggest future research approaches for IIM-ILD.

## Conclusions

Elevated pulmonary FDG uptake, abnormal mediastinal lymph nodes and decreased DLCO% were significantly correlated with the development of RP-ILD in IIM-ILD patients. A DLM model was constructed and initially proved efficient in predicting RP-ILD in these patients. Higher disease activity and complications of bacterial infection were significantly related to unfavourable outcomes. In addition, IIM-ILD patients with higher pulmonary and splenic FDG uptake (which were correlated) showed a tendency to suffer from unfavourable outcomes, indicating a systemic hyperinflammatory status and a possible linkage between the two organs.

## Supplementary Information

**Additional file 1.** Enrollment and groupings of IIM-ILD patients

**Additional file 2.** Univariate logistic regression analyses of RP-ILD in IIM-ILD patients

**Additional file 3.** Multivariate logistic regression analysis of RP-ILD in IIM-ILD patients

**Additional file 4.** Correlation of DLCO%, bilateral lung SUVmean and AML

**Additional file 5.** Correlation of bilateral lung SUVmean, infection, MYOACT score and time gap after RS onset

**Additional file 6.** Multivariate logistic regression analysis of RP-ILD after inclusion of DLM score

**Additional file 7.** Univariate Cox proportional hazards regression analyses of survival in IIM-ILD patients

**Additional file 8.** Multivariate Cox proportional hazards regression analysis of survival in IIM-ILD patients

**Additional file 9.** Comparisons of bilateral lung SUVmean and spleen SUVmean in RP-ILD patients with different survival

**Additional file 10.** Dataset supporting the conclusions of this article

## Data Availability

The dataset supporting the conclusions of this article is presented as Additional file 10 (seen in additional files of this article).

## Abbreviations

ILD: Interstitial lung disease
NSIP: Non-specific interstitial pneumonia
IIM-ILD: Idiopathic-inflammatory-myopathy-related ILD
RP: Rapidly progressive
IPF: Idiopathic pulmonary fibrosis
CTD-ILD: Connective tissue disease-related ILD
COVID-19: Coronavirus disease 2019
FDG: Fluorodeoxyglucose
SSc-ILD: Systemic sclerosis-related ILD
SUV: Standard uptake value
ROI: Region of interest
FAHZJU: The First Affiliated Hospital, Zhejiang University School of Medicine
IRB: Institutional Review Board
EMR: Electronic medical record
ICD-10: International Classification of Diseases, tenth version
DM: Dermatomyositis
PM: Polymyositis
ADM: Amyopathic dermatomyositis
HRCT: High-resolution computed tomography
UIP: Usual interstitial pneumonia
CTD: Connective tissue disease
MYOACT: Myositis Disease Activity Assessment Visual Analogue Scales
PSL: Prednisolone
mPSL: Methylprednisolone
DMARDs: Disease modifying anti-rheumatic drugs
JAK: Janus kinase
IVIG: Intravenous immunoglobulin
FDR: False discovery rate
ROC: Receiver operating characteristic
TLC: Total lung capacity
DLCO%: Percent-predicted diffusing capacity of the lung for carbon monoxide
AUC: Area under the curve
PPV: Positive predictive value
NPV: Negative predictive value
GAP: Gender, age and physiology
