# Supplementary material for: The lungs were on fire: a pilot study of 18F-FDG PET/CT in idiopathic-inflammatory-myopathy-related interstitial lung disease
Source: Arthritis Res Ther. 2021 Jul 23;23:198. doi: 10.1186/s13075-021-02578-9 (PMC8298695; doi:10.1186/s13075-021-02578-9)
Supplement: Supplementary file 1 — Additional file 1. Enrollment and groupings of IIM-ILD patients [file 13075_2021_2578_MOESM1_ESM.docx]

**Additional file 1 Enrollment and groupings of IIM-ILD patients**

IIM-ILD: Idiopathic-inflammatory-myopathy-related interstitial lung disease; RP-ILD: Rapidly progressive interstitial lung disease.

**
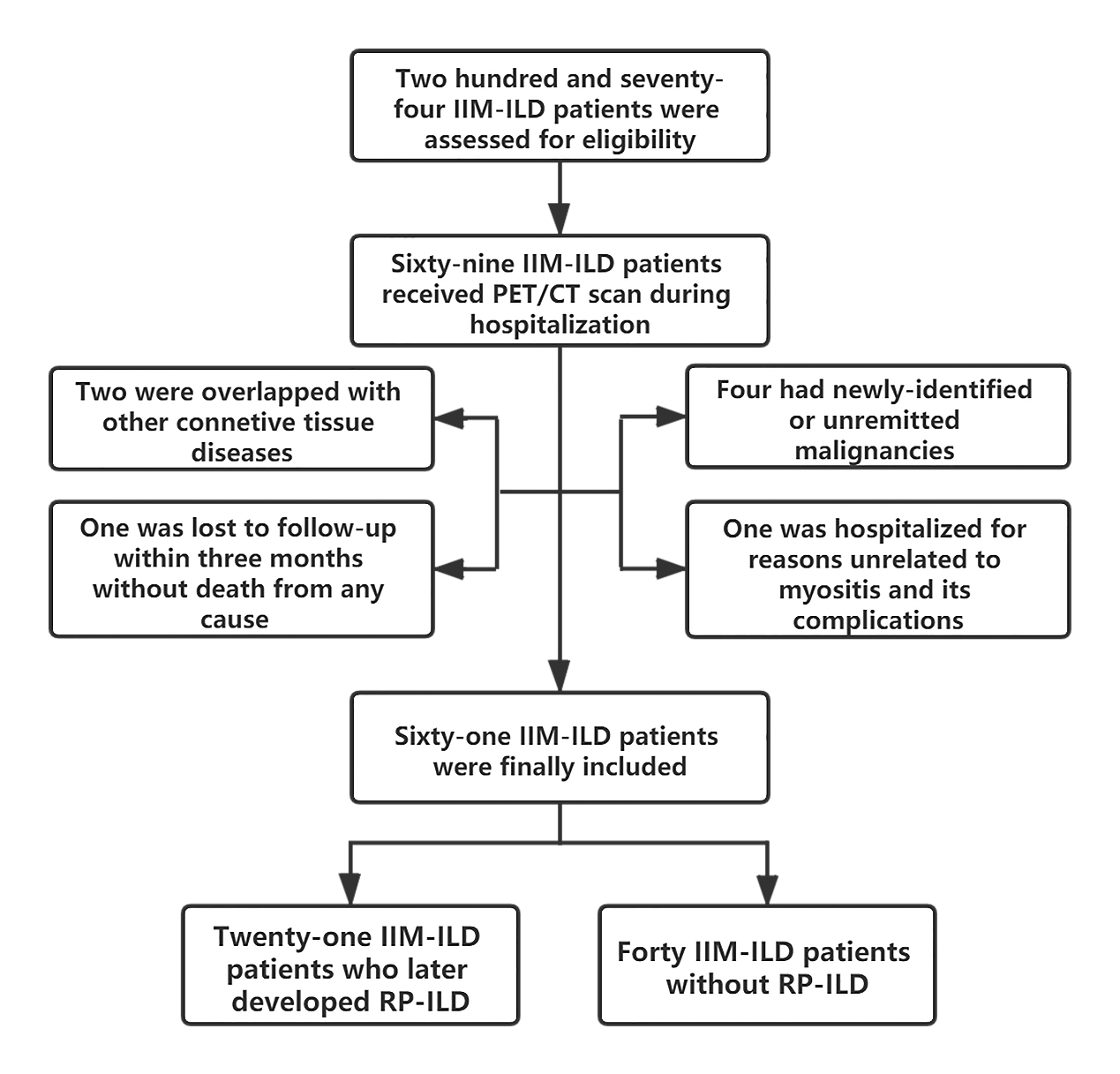
**
