# Supplementary material for: The lungs were on fire: a pilot study of 18F-FDG PET/CT in idiopathic-inflammatory-myopathy-related interstitial lung disease
Source: Arthritis Res Ther. 2021 Jul 23;23:198. doi: 10.1186/s13075-021-02578-9 (PMC8298695; doi:10.1186/s13075-021-02578-9)
Supplement: Supplementary file 2 — Additional file 2. Univariate logistic regression analyses of RP-ILD in IIM-ILD patients [file 13075_2021_2578_MOESM2_ESM.docx]

**Additional file 2 Univariate logistic regression analyses of RP-ILD in IIM-ILD patients**

RP-ILD: Rapidly progressive interstitial lung disease; IIM-ILD: Idiopathic-inflammatory-myopathy-related interstitial lung disease; OR: Odds ratio; CI: Confidence interval; P-adjusted: Adjusted P value after false discovery rate correction; y: years; m: months; NA: Not available; EBV: Epstein-Barr virus; CMV: Cytomegalovirus; UIP pattern: Usual interstitial pneumonia pattern; ESR: Erythrocyte sedimentation rate; CRP: C-reactive protein; ALT: Alaninetransaminase; AST: Aspartate transaminase; LDH: Lactate dehydrogenase; CK: Creatine kinase; MYOACT: Myositis Disease Activity Assessment Visual Analogue Scales; FVC%: Percent-predicted forced vital capacity; FEV1%: Percent-predicted forced expiratory volume in one second; FEV1/FVC: Ratio of FEV1 over FVC; TLC: Total lung capacity; DLCO%: Percent-predicted diffusing capacity of the lung for carbon monoxide; RS: Respiratory symptoms; d: days; FDG: Fluorodeoxyglucose; SUVmean: mean standard uptake value; DMARDs*:* Disease-modifying anti-rheumatic drugs; IVIG: Intravenous immunoglobulin; JAK: Janus kinase; IIM: Idiopathic inflammatory myopathy; DM: dermatomyositis; PM: Polymyositis; ADM: Amyopathic dermatomyositis.

*Time gap after RS onset referred to the time gap between onset of respiratory symptoms (evident feelings of chest distress and shortness of breath) and PET/CT scan.

| **Factors** | **P value** | **OR value** | **95% CI** | **P-adjusted** |
| --- | --- | --- | --- | --- |
| **Age(y)** | **0.349** | **0.978** | **0.932~1.025** | **0.766** |
| **Sex(male/female)** | **0.740** | **0.833** | **0.282~2.454** | **0.968** |
| **Course of disease(m)** | **0.097** | **0.798** | **0.611~1.042** | **0.491** |
| **Duration of diagnosis delay(m)** | **0.225** | **0.787** | **0.535~1.159** | **0.665** |
| **Clinical manifestations or complications** | | | |  |
| **Pulmonary bacterial infection** | **0.032** | **4.500** | **1.138~17.795** | **0.311** |
| **Pulmonary fungal infection** | **0.159** | **2.812** | **0.666~11.878** | **0.564** |
| **Tuberculosis infection** | **NA** | **NA** | **NA** | **NA** |
| **EBV infection** | **0.130** | **2.800** | **0.739~10.609** | **0.529** |
| **CMV infection** | **0.999** | **>100.000** | **0.000~>100.000** | **1.000** |
| **Carcinoma** | **0.259** | **0.283** | **0.032~2.526** | **0.677** |
| **Gastrointestinal hemorrhage** | **0.055** | **9.176** | **0.954~88.303** | **0.416** |
| **UIP pattern** | **0.619** | **1.500** | **0.303~7.432** | **0.911** |
| **Pneumomediastinum** | **0.643** | **1.950** | **0.116~32.837** | **0.911** |
| **Laboratory finding** | | | | |
| **Ferritin(ng/ml)** | **0.285** | **1.000** | **1.000~1.000** | **0.704** |
| **ESR(mm/h)** | **0.432** | **0.992** | **0.972~1.012** | **0.704** |
| **CRP(mg/L)** | **0.252** | **0.989** | **0.971~1.008** | **0.677** |
| **ALT(U/L)** | **0.074** | **0.994** | **0.988~1.001** | **0.436** |
| **AST(U/L)** | **0.140** | **0.998** | **0.995~1.001** | **0.529** |
| **LDH(U/L)** | **0.506** | **0.999** | **0.997~1.001** | **0.819** |
| **CK(U/L)** | **0.208** | **0.999** | **0.998~1.000** | **0.655** |
| **Disease activity** | | | |  |
| **MYOACT score** | **0.007** | **1.223** | **1.056~1.416** | **0.119** |
| **Lung function testing** | | | |  |
| **FVC%(%)** | **0.166** | **0.978** | **0.948~1.009** | **0.564** |
| **FEV1%(%)** | **0.067** | **0.973** | **0.944~1.002** | **0.436** |
| **FEV1/FVC** | **0.913** | **0.792** | **0.012~51.886** | **1.000** |
| **TLC(L)** | **0.054** | **0.571** | **0.323~1.010** | **0.416** |
| **DLCO%(%)** | **0.002** | **0.931** | **0.890~0.974** | **0.045** |
| **^18^F-FDG PET/CT scan findings** | | | | |
| **Time gap after RS onset*(d)** | **0.376** | **0.982** | **0.943~1.022** | **0.776** |
| **Bilateral lung SUVmean** | **0.002** | **>100.000** | **15.909~>100.000** | **0.045** |
| **Abnormal mediastinal lymph node** | **0.002** | **6.000** | **1.889~19.055** | **0.045** |
| **Abnormal hilar lymph node** | **0.021** | **3.789** | **1.220~11.767** | **0.238** |
| **Liver SUVmean** | **0.407** | **1.683** | **0.492~5.757** | **0.776** |
| **Spleen SUVmean** | **0.134** | **2.405** | **0.763~7.578** | **0.529** |
| **Bone marrow SUVmean** | **0.397** | **1.640** | **0.522~5.150** | **0.776** |
| **Cardiac SUVmean** | **0.993** | **0.999** | **0.717~1.391** | **1.000** |
| **Esophagus SUVmean** | **0.333** | **1.570** | **0.630~3.915** | **0.755** |
| **Stomach SUVmean** | **0.409** | **1.882** | **0.420~8.436** | **0.776** |
| **Small intestine SUVmean** | **0.876** | **1.141** | **0.218~5.968** | **1.000** |
| **Colon and rectum SUVmean** | **0.313** | **1.752** | **0.590~5.202** | **0.734** |
| **Bilateral cerebellum SUVmean** | **0.860** | **0.968** | **0.676~1.387** | **1.000** |
| **Bilateral trapezius SUVmean** | **0.938** | **0.930** | **0.151~5.712** | **1.000** |
| **Bilateral deltoid SUVmean** | **0.726** | **0.767** | **0.173~3.395** | **0.968** |
| **Bilateral biceps SUVmean** | **0.482** | **0.532** | **0.091~3.091** | **0.819** |
| **Bilateral ilioposas SUVmean** | **0.847** | **0.864** | **0.196~3.809** | **1.000** |
| **Bilateral gluteus maximus SUVmean** | **0.461** | **1.926** | **0.337~11.022** | **0.813** |
| **Bilateral gluteus medius SUVmean** | **0.466** | **0.493** | **0.074~3.298** | **0.813** |
| **Bilateral quadriceps SUVmean** | **0.769** | **0.759** | **0.121~4.750** | **0.987** |
| **Myositis-specific antibodies & Myositis-associated antibodies** | | | |  |
| **Anti-MDA5** | **0.019** | **3.792** | **1.249~11.510** | **0.238** |
| **Anti-PL-7** | **0.101** | **4.471** | **0.746~26.807** | **0.491** |
| **Anti-PL-12** | **0.643** | **1.950** | **0.116~32.837** | **0.911** |
| **Anti-EJ** | **1.000** | **0.000** | **0.000~>100.000** | **1.000** |
| **Anti-OJ** | **1.000** | **0.000** | **0.000~>100.000** | **1.000** |
| **Anti-Jo-1** | **0.785** | **1.298** | **0.200~8.447** | **0.989** |
| **Anti-TIF1γ** | **0.684** | **0.617** | **0.060~6.323** | **0.930** |
| **Anti-Mi-2α** | **0.999** | **0.000** | **0.000~>100.000** | **1.000** |
| **Anti-Mi-2β** | **0.999** | **0.000** | **0.000~>100.000** | **1.000** |
| **Anti-SAE1** | **0.999** | **0.000** | **0.000~>100.000** | **1.000** |
| **Anti-NXP2** | **0.259** | **0.283** | **0.032~2.526** | **0.677** |
| **Anti-SRP** | **0.967** | **0.950** | **0.081~11.128** | **1.000** |
| **Anti-Ku** | **0.643** | **1.950** | **0.116~32.837** | **0.911** |
| **Anti-PM-Scl75** | **0.967** | **0.950** | **0.081~11.128** | **1.000** |
| **Anti-PM-Scl100** | **NA** | **NA** | **NA** | **NA** |
| **Anti-Ro-52** | **0.077** | **2.706** | **0.898~8.152** | **0.436** |
| **Therapies** | | | |  |
| **Steroid monotherapy** | **0.612** | **0.743** | **0.236~2.342** | **0.911** |
| **Steroid+DMARDs** | **0.290** | **0.553** | **0.184~1.659** | **0.704** |
| [**Steroid+IVIG**](http://www.baidu.com/link?url=_srwKTXKnet8GknUvvs0xyTJdpfNOQtIDWHWhe_U5wypEldT9OPh2gCg3LsSDR-5CpyLTLOBAy4p4ov8wle8F6_YWPs4sPX-lyXINgDKaDW) | **0.505** | **2.000** | **0.261~15.318** | **0.819** |
| [**Steroid+DMARDs+IVIG**](http://www.baidu.com/link?url=uciYHxddnq2QF5VJVWJRCy7Q7nEAXlzzmiKvgGzZkrPg72XHW0qrc1acnFRmU-CtSPSZqd_rW-WBKuZFe0OpuS_h9gOsjyItDqvwfb_UtbdGjXJvU0FWCCPVF1qaXYLk) | **0.619** | **1.500** | **0.303~7.432** | **0.911** |
| **Steroid+JAK inhibitor** | **0.116** | **6.500** | **0.632~66.878** | **0.526** |
| **IIM subtypes** | | | |  |
| **DM** | **0.662** | **0.782** | **0.260~2.354** | **0.919** |
| **PM** | **0.411** | **0.496** | **0.093~2.635** | **0.776** |
| **ADM** | **0.212** | **2.267** | **0.627~8.188** | **0.655** |
