# Supplementary material for: The lungs were on fire: a pilot study of 18F-FDG PET/CT in idiopathic-inflammatory-myopathy-related interstitial lung disease
Source: Arthritis Res Ther. 2021 Jul 23;23:198. doi: 10.1186/s13075-021-02578-9 (PMC8298695; doi:10.1186/s13075-021-02578-9)
Supplement: Supplementary file 3 — Additional file 3. Multivariate logistic regression analysis of RP-ILD in IIM-ILD patients [file 13075_2021_2578_MOESM3_ESM.docx]

**Additional file 3 Multivariate logistic regression analysis of RP-ILD in IIM-ILD patients**

RP-ILD: Rapidly progressive interstitial lung disease; IIM-ILD: Idiopathic-inflammatory-myopathy-related interstitial lung disease; OR: Odds ratio; CI: Confidence interval; DLCO%: Percent-predicted diffusing capacity of the lung for carbon monoxide; SUVmean: mean standard uptake value.

| **Factors** | **P value** | **OR value** | **95% CI** |
| --- | --- | --- | --- |
| **DLCO%** | **0.003** | **0.898** | **0.837~0.964** |
| **Bilateral lung SUVmean** | **0.001** | **>100.000** | **81.904~>100.000** |
| **Abnormal mediastinal lymph node** | **0.013** | **7.735** | **1.527~39.183** |
