# Supplementary material for: The lungs were on fire: a pilot study of 18F-FDG PET/CT in idiopathic-inflammatory-myopathy-related interstitial lung disease
Source: Arthritis Res Ther. 2021 Jul 23;23:198. doi: 10.1186/s13075-021-02578-9 (PMC8298695; doi:10.1186/s13075-021-02578-9)
Supplement: Supplementary file 4 — Additional file 4. Correlation of DLCO%, bilateral lung SUVmean and AML [file 13075_2021_2578_MOESM4_ESM.docx]

**Additional file 4 Correlation of DLCO%, bilateral lung SUVmean and AML**

A. Correlation between DLCO% and bilateral lung SUVmean

B. Correlation between AML and bilateral lung SUVmean

C. Correlation between AML and DLCO%

DLCO%: Percent-predicted diffusing capacity of the lung for carbon monoxide; SUVmean: mean standard uptake value; AML: Abnormal mediastinal lymph node; NML: Normal mediastinal lymph node.

**
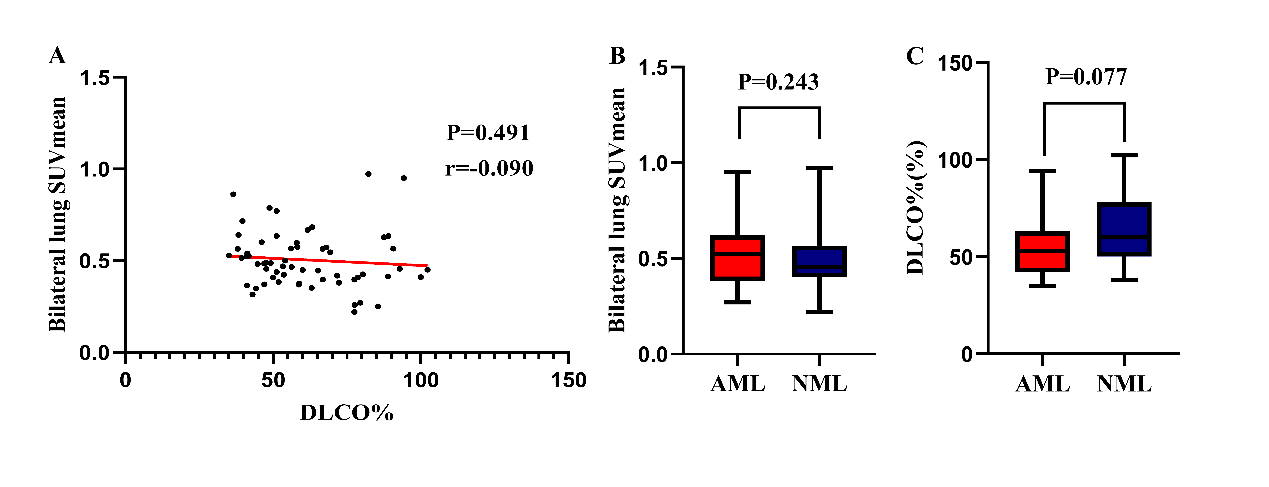
**
