# Supplementary material for: The lungs were on fire: a pilot study of 18F-FDG PET/CT in idiopathic-inflammatory-myopathy-related interstitial lung disease
Source: Arthritis Res Ther. 2021 Jul 23;23:198. doi: 10.1186/s13075-021-02578-9 (PMC8298695; doi:10.1186/s13075-021-02578-9)

**Additional file 5 Correlation of bilateral lung SUVmean, infection, MYOACT score and time gap after RS onset**

A. Correlation between bilateral lung SUVmean and pulmonary bacterial infection

B. Correlation between bilateral lung SUVmean and pulmonary fungal infection

C. Correlation between bilateral lung SUVmean and MYOACT score

D. Correlation between bilateral lung SUVmean and time gap after RS onset

SUVmean: mean standard uptake value; MYOACT: Myositis Disease Activity Assessment Visual Analogue Scales; Time gap after RS onset: the time gap between onset of respiratory symptoms (evident feelings of chest distress and shortness of breath) and PET/CT scan.


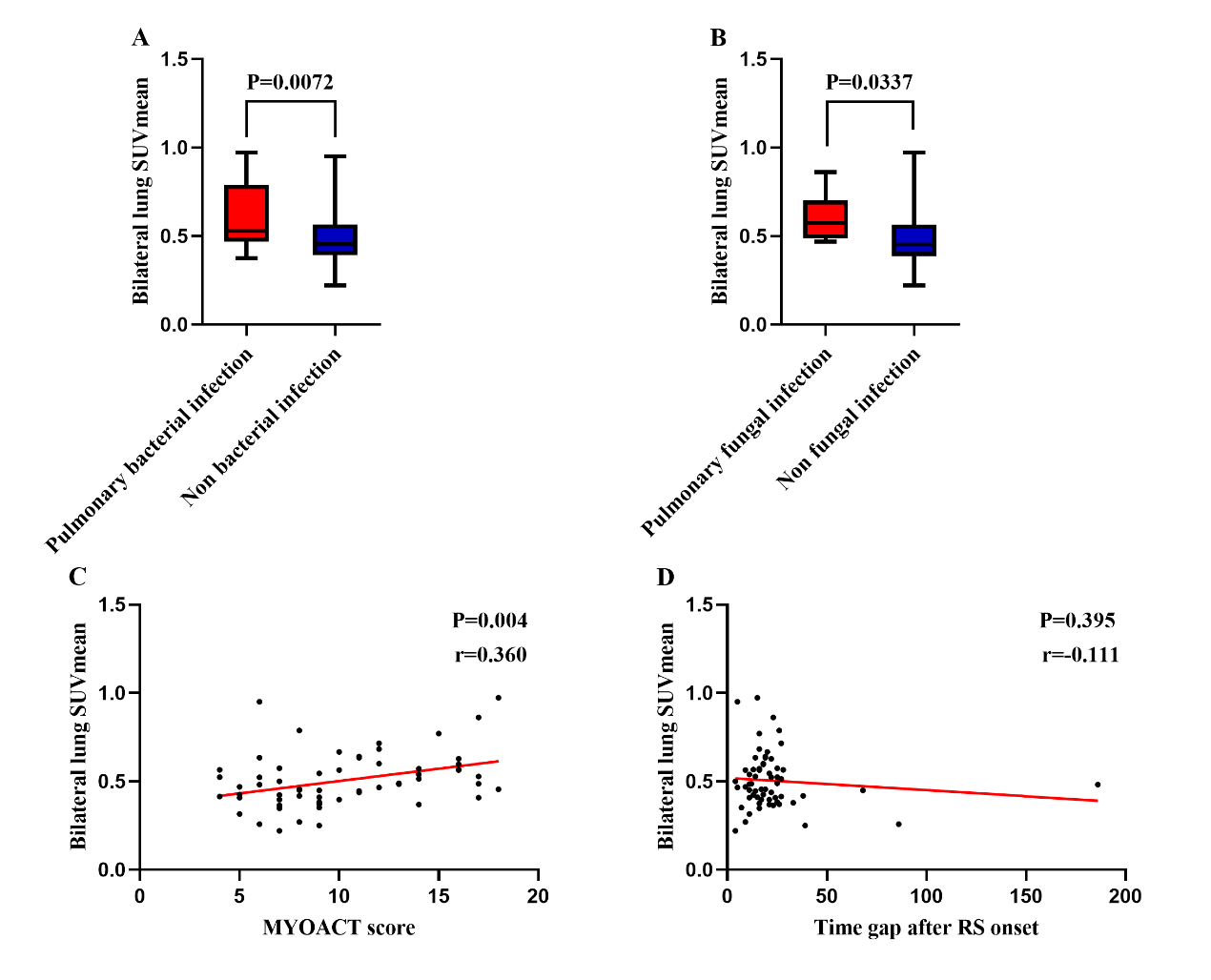

Supplement: Supplementary file 5 — Additional file 5. Correlation of bilateral lung SUVmean, infection, MYOACT score and time gap after RS onset [file 13075_2021_2578_MOESM5_ESM.docx]
