# Supplementary material for: The lungs were on fire: a pilot study of 18F-FDG PET/CT in idiopathic-inflammatory-myopathy-related interstitial lung disease
Source: Arthritis Res Ther. 2021 Jul 23;23:198. doi: 10.1186/s13075-021-02578-9 (PMC8298695; doi:10.1186/s13075-021-02578-9)
Supplement: Supplementary file 6 — Additional file 6. Multivariate logistic regression analysis of RP-ILD after inclusion of DLM score [file 13075_2021_2578_MOESM6_ESM.docx]

**Additional file 6 Multivariate logistic regression analysis of RP-ILD after inclusion of DLM score**

| **Factors** | **P value** | **OR value** | **95% CI** |
| --- | --- | --- | --- |
| **DLM score** | **<0.001** | **13.184** | **3.715~46.787** |
