# Supplementary material for: The lungs were on fire: a pilot study of 18F-FDG PET/CT in idiopathic-inflammatory-myopathy-related interstitial lung disease
Source: Arthritis Res Ther. 2021 Jul 23;23:198. doi: 10.1186/s13075-021-02578-9 (PMC8298695; doi:10.1186/s13075-021-02578-9)
Supplement: Supplementary file 7 — Additional file 7. Univariate Cox proportional hazards regression analyses of survival in IIM-ILD patients [file 13075_2021_2578_MOESM7_ESM.docx]

**Additional file 7 Univariate Cox proportional hazards regression analyses of survival in IIM-ILD patients**

IIM-ILD: Idiopathic-inflammatory-myopathy-related interstitial lung disease; HR: Hazard ratio; CI: Confidence interval; P-adjusted: Adjusted P value after false discovery rate correction; y: years; m: months; NA: Not available; EBV: Epstein-Barr virus; CMV: Cytomegalovirus; UIP pattern: Usual interstitial pneumonia pattern; RP-ILD: Rapidly progressive interstitial lung disease; ESR: Erythrocyte sedimentation rate; CRP: C-reactive protein; ALT: Alaninetransaminase; AST: Aspartate transaminase; LDH: Lactate dehydrogenase; CK: Creatine kinase; MYOACT: Myositis Disease Activity Assessment Visual Analogue Scales; FVC%: Percent-predicted forced vital capacity; FEV1%: Percent-predicted forced expiratory volume in one second; FEV1/FVC: Ratio of FEV1 over FVC; TLC: Total lung capacity; DLCO%: Percent-predicted diffusing capacity of the lung for carbon monoxide; RS: Respiratory symptoms; d: days; FDG: Fluorodeoxyglucose; SUVmean: mean standard uptake value; DMARDs*:* Disease-modifying anti-rheumatic drugs; IVIG: Intravenous immunoglobulin; JAK: Janus kinase; IIM: Idiopathic inflammatory myopathy; DM: dermatomyositis; PM: Polymyositis; ADM: Amyopathic dermatomyositis.

*Time gap after RS onset referred to the time gap between onset of respiratory symptoms (evident feelings of chest distress and shortness of breath) and PET/CT scan.

| **Factors** | **P value** | **HR value** | **95% CI** | **P-adjusted** |
| --- | --- | --- | --- | --- |
| **Age(y)** | **0.748** | **1.006** | **0.970~1.043** | **0.922** |
| **Sex(male/female)** | **0.893** | **1.057** | **0.469~2.381** | **0.963** |
| **Course of disease(m)** | **0.219** | **0.888** | **0.734~1.074** | **0.839** |
| **Duration of diagnosis delay(m)** | **0.099** | **0.744** | **0.524~1.058** | **0.621** |
| **Clinical manifestations or complications** | | | |  |
| **Pulmonary bacterial infection** | **<0.001** | **4.576** | **1.998~10.533** | **<0.001** |
| **Pulmonary fungal infection** | **0.360** | **1.588** | **0.590~4.276** | **0.886** |
| **Tuberculosis infection** | **NA** | **NA** | **NA** | **NA** |
| **EBV infection** | **0.671** | **1.266** | **0.427~3.752** | **0.910** |
| **CMV infection** | **0.453** | **2.169** | **0.288~16.355** | **0.897** |
| **Carcinoma** | **0.612** | **1.323** | **0.449~3.898** | **0.910** |
| **Gastrointestinal hemorrhage** | **0.179** | **2.317** | **0.681~7.887** | **0.839** |
| **UIP pattern** | **0.989** | **1.008** | **0.300~3.395** | **0.989** |
| **RP-ILD** | **0.008** | **3.096** | **1.350~7.102** | **0.110** |
| **Pneumomediastinum** | **0.577** | **0.047** | **0.000~>100.000** | **0.910** |
| **Laboratory finding** | | | | |
| **Ferritin(ng/ml)** | **0.385** | **1.000** | **1.000~1.000** | **0.886** |
| **ESR(mm/h)** | **0.225** | **0.990** | **0.974~1.006** | **0.839** |
| **CRP(mg/L)** | **0.588** | **0.997** | **0.986~1.008** | **0.910** |
| **ALT(U/L)** | **0.492** | **1.001** | **0.999~1.003** | **0.897** |
| **AST(U/L)** | **0.068** | **1.001** | **1.000~1.002** | **0.469** |
| **LDH(U/L)** | **0.215** | **1.001** | **1.000~1.002** | **0.839** |
| **CK(U/L)** | **0.635** | **1.000** | **0.999~1.000** | **0.910** |
| **Disease activity** | | | |  |
| **MYOACT score** | **<0.001** | **1.338** | **1.176~1.523** | **<0.001** |
| **Lung function testing** | | | |  |
| **FVC%(%)** | **0.341** | **0.989** | **0.966~1.012** | **0.886** |
| **FEV1%(%)** | **0.231** | **0.987** | **0.967~1.008** | **0.839** |
| **FEV1/FVC** | **0.727** | **1.627** | **0.106~24.971** | **0.912** |
| **TLC(L)** | **0.066** | **0.684** | **0.456~1.026** | **0.469** |
| **DLCO%(%)** | **0.016** | **0.965** | **0.938~0.994** | **0.158** |
| **^18^F-FDG PET/CT scan findings** | | | | |
| **Time gap after RS onset*(d)** | **0.494** | **0.990** | **0.960~1.020** | **0.897** |
| **Bilateral lung SUVmean** | **0.007** | **27.751** | **2.509~>100.000** | **0.110** |
| **Abnormal mediastinal lymph node** | **0.399** | **1.416** | **0.630~3.182** | **0.888** |
| **Abnormal hilar lymph node** | **0.554** | **1.285** | **0.560~2.951** | **0.910** |
| **Liver SUVmean** | **0.719** | **1.174** | **0.492~2.801** | **0.912** |
| **Spleen SUVmean** | **0.008** | **2.847** | **1.309~6.193** | **0.110** |
| **Bone marrow SUVmean** | **0.620** | **1.251** | **0.516~3.030** | **0.910** |
| **Cardiac SUVmean** | **0.966** | **1.006** | **0.766~1.321** | **0.980** |
| **Esophagus SUVmean** | **0.490** | **1.284** | **0.632~2.608** | **0.897** |
| **Stomach SUVmean** | **0.954** | **0.961** | **0.244~3.785** | **0.980** |
| **Small intestine SUVmean** | **0.349** | **1.800** | **0.526~6.167** | **0.886** |
| **Colon and rectum SUVmean** | **0.314** | **1.485** | **0.688~3.205** | **0.867** |
| **Bilateral cerebellum SUVmean** | **0.854** | **0.974** | **0.733~1.293** | **0.963** |
| **Bilateral trapezius SUVmean** | **0.688** | **1.277** | **0.387~4.216** | **0.910** |
| **Bilateral deltoid SUVmean** | **0.887** | **1.087** | **0.342~3.456** | **0.963** |
| **Bilateral biceps SUVmean** | **0.294** | **0.482** | **0.123~1.882** | **0.867** |
| **Bilateral ilioposas SUVmean** | **0.207** | **2.018** | **0.679~6.003** | **0.839** |
| **Bilateral gluteus maximus SUVmean** | **0.699** | **1.262** | **0.388~4.103** | **0.910** |
| **Bilateral gluteus medius SUVmean** | **0.890** | **1.096** | **0.301~3.990** | **0.963** |
| **Bilateral quadriceps SUVmean** | **0.699** | **1.311** | **0.333~5.160** | **0.910** |
| **Myositis-specific antibodies & Myositis-associated antibodies** | | | |  |
| **Anti-MDA5** | **0.057** | **2.223** | **0.975~5.067** | **0.469** |
| **Anti-PL-7** | **0.311** | **0.355** | **0.048~2.634** | **0.867** |
| **Anti-PL-12** | **0.881** | **1.167** | **0.155~8.800** | **0.963** |
| **Anti-EJ** | **0.613** | **0.048** | **0.000~>100.000** | **0.910** |
| **Anti-OJ** | **0.689** | **0.048** | **0.000~>100.000** | **0.910** |
| **Anti-Jo-1** | **0.941** | **0.946** | **0.222~4.032** | **0.980** |
| **Anti-TIF1γ** | **0.942** | **1.073** | **0.251~4.588** | **0.980** |
| **Anti-Mi-2α** | **0.485** | **0.046** | **0.000~>100.000** | **0.897** |
| **Anti-Mi-2β** | **0.376** | **0.044** | **0.000~44.397** | **0.886** |
| **Anti-SAE1** | **0.626** | **0.692** | **0.158~3.034** | **0.910** |
| **Anti-NXP2** | **0.209** | **0.277** | **0.037~2.055** | **0.839** |
| **Anti-SRP** | **0.111** | **2.707** | **0.796~9.206** | **0.638** |
| **Anti-Ku** | **0.881** | **1.167** | **0.155~8.800** | **0.963** |
| **Anti-PM-Scl75** | **0.467** | **1.719** | **0.400~7.384** | **0.897** |
| **Anti-PM-Scl100** | **NA** | **NA** | **NA** | **NA** |
| **Anti-Ro-52** | **0.302** | **1.536** | **0.680~3.468** | **0.867** |
| **Therapies** | | | |  |
| **Steroid monotherapy** | **0.298** | **0.624** | **0.257~1.517** | **0.867** |
| **Steroid+DMARDs** | **0.655** | **0.828** | **0.362~1.895** | **0.910** |
| [**Steroid+IVIG**](http://www.baidu.com/link?url=_srwKTXKnet8GknUvvs0xyTJdpfNOQtIDWHWhe_U5wypEldT9OPh2gCg3LsSDR-5CpyLTLOBAy4p4ov8wle8F6_YWPs4sPX-lyXINgDKaDW) | **0.015** | **4.810** | **1.365~16.949** | **0.158** |
| [**Steroid+DMARDs+IVIG**](http://www.baidu.com/link?url=uciYHxddnq2QF5VJVWJRCy7Q7nEAXlzzmiKvgGzZkrPg72XHW0qrc1acnFRmU-CtSPSZqd_rW-WBKuZFe0OpuS_h9gOsjyItDqvwfb_UtbdGjXJvU0FWCCPVF1qaXYLk) | **0.306** | **1.753** | **0.598~5.139** | **0.867** |
| **Steroid+JAK inhibitor** | **0.827** | **0.799** | **0.106~6.004** | **0.963** |
| **IIM subtypes** | | | |  |
| **DM** | **0.605** | **1.252** | **0.534~2.936** | **0.910** |
| **PM** | **0.880** | **1.086** | **0.369~3.194** | **0.963** |
| **ADM** | **0.470** | **0.673** | **0.230~1.971** | **0.897** |
