# Supplementary material for: The lungs were on fire: a pilot study of 18F-FDG PET/CT in idiopathic-inflammatory-myopathy-related interstitial lung disease
Source: Arthritis Res Ther. 2021 Jul 23;23:198. doi: 10.1186/s13075-021-02578-9 (PMC8298695; doi:10.1186/s13075-021-02578-9)
Supplement: Supplementary file 8 — Additional file 8. Multivariate Cox proportional hazards regression analysis of survival in IIM-ILD patients [file 13075_2021_2578_MOESM8_ESM.docx]

**Additional file 8 Multivariate Cox proportional hazards regression analysis of survival in IIM-ILD patients**

IIM-ILD: Idiopathic-inflammatory-myopathy-related interstitial lung disease; HR: Hazard ratio; CI: Confidence interval; MYOACT: Myositis Disease Activity Assessment Visual Analogue Scales.

| **Factors** | **P value** | **HR value** | **95% CI** |
| --- | --- | --- | --- |
| **Pulmonary bacterial infection** | **0.013** | **3.021** | **1.261~7.233** |
| **MYOACT score** | **<0.001** | **1.301** | **1.140~1.483** |
