# Supplementary material for: The lungs were on fire: a pilot study of 18F-FDG PET/CT in idiopathic-inflammatory-myopathy-related interstitial lung disease
Source: Arthritis Res Ther. 2021 Jul 23;23:198. doi: 10.1186/s13075-021-02578-9 (PMC8298695; doi:10.1186/s13075-021-02578-9)
Supplement: Supplementary file 9 — Additional file 9. Comparisons of bilateral lung SUVmean and spleen SUVmean in RP-ILD patients with different survival [file 13075_2021_2578_MOESM9_ESM.docx]

**Additional file 9 Comparisons of bilateral lung SUVmean and spleen SUVmean in RP-ILD patients with different survival**

A. Comparison of bilateral lung SUVmean in RP-ILD patients who died within three months or survived beyond this threshold

B. Comparison of spleen SUVmean in RP-ILD patients who died within three months or survived beyond this threshold

SUVmean: mean standard uptake value; RP-ILD: Rapidly progressive interstitial lung disease.

**
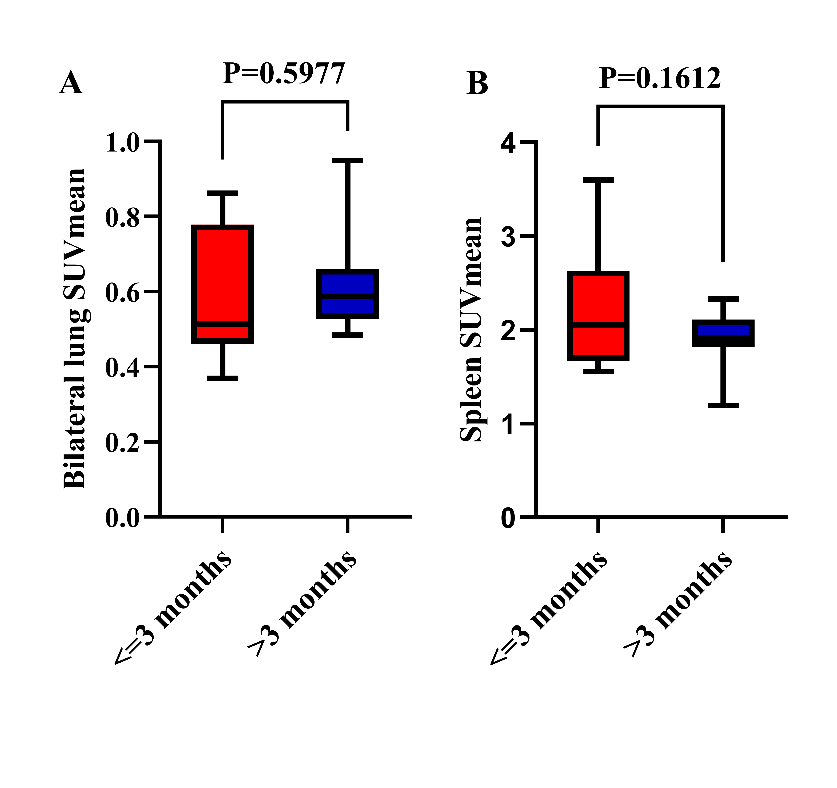
**
